# Seesaw nystagmus with internuclear ophthalmoplegia from bilateral dorsomedial pons and left thalamus infarction: a case report

**DOI:** 10.1186/s13256-019-2269-3

**Published:** 2019-11-29

**Authors:** Qian Zhang, Jian Li

**Affiliations:** Department of Neurology, the First Affiliated Hospital of Jinzhou Medical University, No. 2, Section 5, People Street, Jinzhou, 121000 Liaoning China

**Keywords:** Seesaw nystagmus, Internuclear ophthalmoplegia, Interstitial nucleus of Cajal, Bilateral dorsomedial pons infarction

## Abstract

**Background:**

We describe for the first time the clinical features and mechanisms of a bilateral dorsomedial pons and left thalamus infarction with seesaw nystagmus and internuclear ophthalmoplegia.

**Case presentation:**

A 62-year-old Chinese man was hospitalized for sudden-onset dizziness, diplopia, and gait disturbance. A neurological examination revealed seesaw nystagmus and internuclear ophthalmoplegia. Magnetic resonance imaging disclosed an acute infarction confined to the bilateral dorsomedial pons and left thalamus. Subsequently, 2 weeks of antithrombotic therapy led to an improvement in his symptoms.

**Conclusions:**

This case illustrates that the acute onset of seesaw nystagmus and internuclear ophthalmoplegia accompanied by risk factors for cerebrovascular diseases are highly suggestive of brainstem infarction.

## Introduction

Seesaw nystagmus (SSN) is a rare ocular motor disorder characterized by cyclic eye movements with a conjugate torsional component and a dissociated vertical component. While one eye elevates and intorts, the other eye depresses and extorts; the movement pattern is reversed in the remaining half of the cycle [1]. Internuclear ophthalmoplegia (INO) is characterized by adduction paresis of the ipsilesional eye and dissociated abducting nystagmus of the contralesional eye on attempted gaze to the contralesional side. INO is a complex ocular motility disorder caused by damage to the medial longitudinal fasciculus (MLF). SSN has been reported rarely in association with INO to the best of our knowledge. Here, we report the case of a patient with SSN and INO from bilateral dorsomedial pons and left thalamus infarction. These signs seem to be caused by selective damage to the excitatory fibers originating in the contralateral vertical semicircular canal. The patient had various risk factors of cerebrovascular disease. Therefore, we propose that the acute onset of this constellation of signs is highly suggestive of pontine infarction.

## Case presentation

A 62-year-old man, right-handed, Chinese man, an entrepreneur, was admitted to our department for a day with sudden-onset dizziness, diplopia, and gait disturbance. He had a history of hypertension for 10 years and was currently taking the orally administered antihypertensive drug telmisartan. In addition, he had been diagnosed as having diabetes 4 years ago and was currently taking metformin. He suffered from cerebral infarction in 2009 and 2010 but had no residual neurological deficits. Beyond the above mentioned, he denied any history of trauma and infectious diseases. He had been smoking two packs of cigarettes per day and drinking alcohol occasionally for 20 years. He quit tobacco smoking and drinking alcohol 4 years ago.

His physical examination on admission showed the following. His temperature was 37 ºC. His pulse was 95 beats every minute. His respiratory rate was 21 times every minute. His blood pressure was 120 over 80. The physical examination showed that his thorax was symmetrical without deformity. There was no tenderness or varicose veins in his chest wall. In addition, bilateral respiratory sounds were clear, without murmur. The appearance of his abdomen was flat and symmetric. His abdominal breathing was normal. Furthermore, a gastrointestinal and peristaltic wave was not seen. No abnormal liver, gallbladder, pancreas, spleen, and kidney were found on palpation. He had no deformity of limbs, no abnormal joint activity, no tenderness and atrophy of muscles, and no varicose veins of lower limbs. No edema was found in both lower limbs and the pulsation of the dorsal artery of both his feet was normal. His complete blood count tests revealed an increased neutrophil count of 7.28(10^9^/l) and a normal red blood cell count, platelet count, and normal hemoglobin count. Laboratory results showed that his fasting blood glucose was 10.88 mmol/l (reference 3.89–6.11 mmol/l). In addition, his glycosylated hemoglobin ratio was 9% (reference 4–6%). Urine glucose results showed 4+. The results of alanine aminotransferase and glutamic oxaloacetic aminotransferase tests were normal. No abnormality of human immunodeficiency virus antigen-antibody, *Treponema pallidum*-specific antibody, and hepatitis C virus antibody were found. There was no inherited disease in his family.

**Fig. 1:**
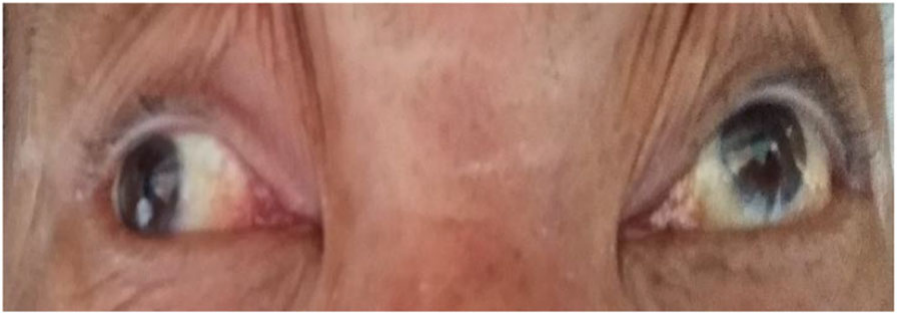
The patient showed adduction paresis in the left eye and dissociated abducting paresis in the right eye during rightward gaze

**Fig. 2:**
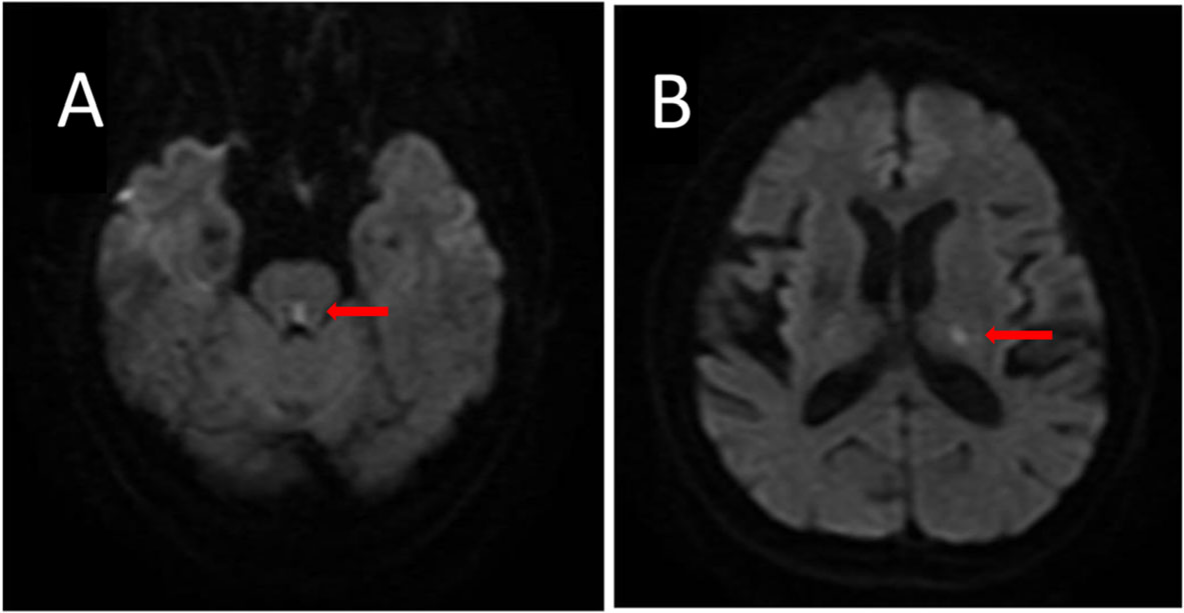
Diffusion-weighted brain magnetic resonance imaging shows two infarctions in bilateral dorsomedial pons (red arrow in **a**) and left thalamus infarction (red arrow in **b**)

**Fig. 3:**
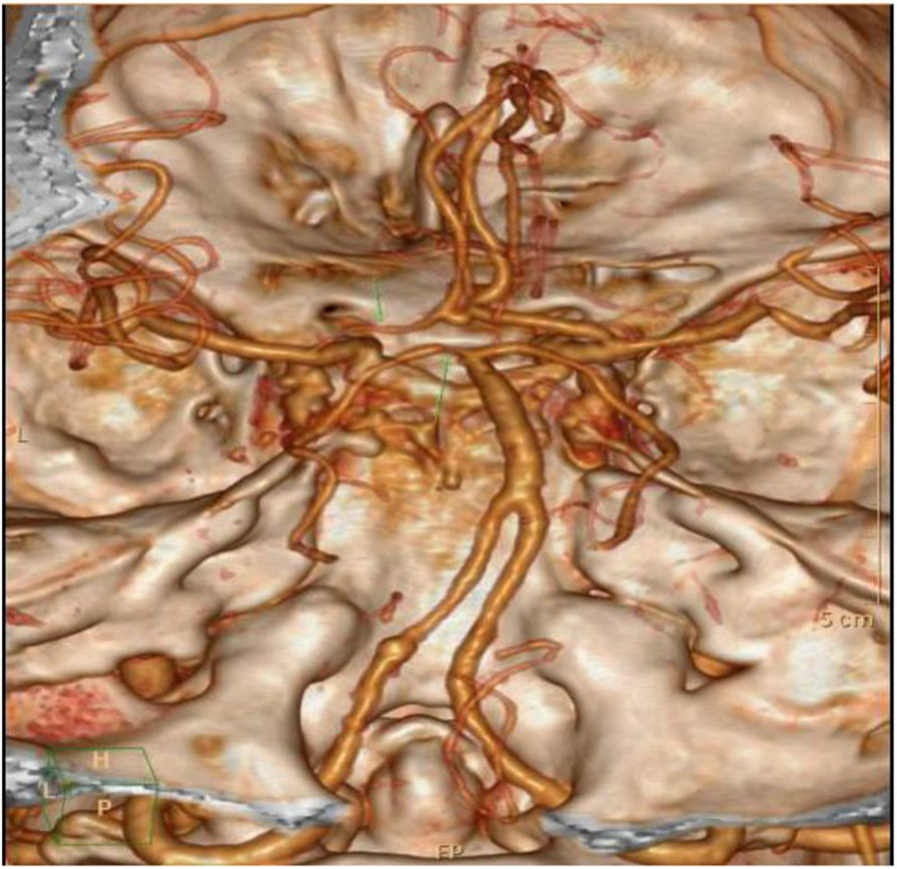
Computerized tomography angiography showed the left anterior cerebral artery was slender and the ipsilateral posterior cerebral artery had stenosis (green arrow)


On admission, he could hardly open his eyes owing to severe oscillopsia. A neurological examination revealed extorsional downbeat nystagmus in the left eye and intorsional upbeat nystagmus in the right eye, with the horizontal component to the left side in the primary position (Additional file 1: Video S1) which is consistent with SSN. The magnitude of nystagmus was pronounced in the right gaze but decreased with a downward gaze. Our patient showed adduction paresis in the left eye and dissociated abducting paresis in the right eye during rightward gaze (Fig. 1). The leftward saccades also disclosed abducting lag in the left eye. The rest of the neurologic findings were, otherwise, unremarkable. Saccadic oscillations and SSN were consistently present during primary and eccentric gaze holding. His gait was extremely impaired due to the perception of oscillations. Horizontal head impulse tests were normal in both directions. Except for hyperglycemia as well as hypertriglyceridemia, the remaining laboratory evaluation was normal. An electronystagmogram (ENG) revealed SSN and a Bárány test was negative (Fig. 2). As we all know, an infarction of pontine corresponds to the anatomic location of the MLF. Diffusion-weighted (DW) brain magnetic resonance imaging (MRI) disclosed an acute infarction confined to the bilateral dorsomedial pons and left thalamus. Computed tomography angiography (CTA) showed the left anterior cerebral artery (ACA) was slender and the ipsilateral posterior cerebral artery (PCA) had stenosis (Fig. 3). Subsequently, 2 weeks of antithrombotic therapy led to an improvement in our patient’s dizziness as well as nystagmus. The saccades were better characterized when his gaze holding had improved. On discharge, he reported no dizziness but had certain degrees of adduction weakness. After 6 months of follow-up, there was no recurrence of dizziness symptoms and a nervous system physical examination basically returned to normal.

**Additional file 1: Video S1.** Video of the signs of this patient with seesaw nystagmus.


## Discussion and conclusion

Our patient presented with SSN accompanying INO. Moreover, the infarction of pontine corresponded to the anatomic location of the lateral MLF, which was rare to see; that is the unusual point in this case. Most importantly, our case illustrates that the acute onset of this constellation of signs is highly suggestive of pontine infarction.

SSN can be seen in disorders of visual pathways, especially the optic chiasm. SSN has been reported in the literature with parasellar lesions such as pituitary macroadenoma [2], brainstem infarcts, multiple sclerosis (MS), and congenital absence of the optic chiasma. The interstitial nucleus of Cajal (INC) is located in the anterolateral superior colliculus and regulates the rotation of the eyeball. The pathophysiology of SSN is considered related to the disturbance of the graviceptive pathway between the vestibular nucleus and INC. The INO is characterized by adduction paresis of the ipsilesional eye and dissociated abducting nystagmus of the contralesional eye on attempted gaze to the contralesional side, a complex ocular motility disorder caused by damage to the MLF. SSN is rarely associated with INO. Our patient, who had many cerebrovascular risk factors, had SSN and INO as sole manifestations of infarction in the bilateral dorsomedial pons; hence, an acute ischemic stroke affecting the pontine junction was strongly suspected at the beginning. Subsequent magnetic resonance examination results confirmed this assumption. Trial of ORG 10172 in Acute Stroke Treatment (TOAST) classification of this case is considered to be large-artery atherosclerosis. The region of the pons in our report contained the MLF that extended through the brainstem and lay near the midline just ventral to the fourth ventricle in the pons [3]. The neural integrators (NI) are localized in the brainstem, adjacent to the extraocular motor neuron nuclei used to stabilize eye position in eccentric position for comfortable single vision. Hence, general dysfunction of NI will lead to symptoms such as blurred vision, vestibular imbalance, and vertigo [4]. INC is a small collection of neurons located adjacent to MLF, which function as NI for vertical and torsional eye movements. Visual, vestibular, and ocular motor interaction also occurs in the INC. It is a significant unit of the “eye movement neural integrator” which combines ocular velocity signals and encodes them into position commands [5]. Kim and Lee [6] reported the case of a patient with bilateral INO and vertical gaze-evoked nystagmus (GEM) as sole manifestations of paramedian pontine infarction. They suggested that vertical gaze-holding failure due to the involvement of the MLF and/or paramedian tract (PMT) neurons might be related to vertical GEM. Choi *et al.*’s [7] findings demonstrated that INO can be accompanied by various oculomotor abnormalities including SSN. However, the previous case had unilateral MLF whereas our present case had bilateral MLF that is rare to see. Some researchers indicated that SSN was ascribed to unilateral inactivation of INC (the torsional eye-velocity integrator), with sparing of the torsional fast-phase generator, the rostral interstitial nucleus of MLF [8]. Bilateral dorsomedial infarcts of the pons are uncommon and the neuro-ophthalmic presentation can be quite variable. The mechanisms are still unclear. We suspect that MLF lesions may lead to different types of dissociated torsional–vertical nystagmus, depending on the patterns involving the pathways from contralateral vertical semicircular canals and probably from the otoliths of the contralateral labyrinth. The association of the SSN and INO is rare; whenever present, the etiology was demyelinating or neoplastic. However, the constellation of acute onset of SSN and INO in a patient with the risk factors for cerebrovascular diseases highlighted the likelihood of acute brainstem stroke.

## Data Availability

Not applicable.

## Abbreviations

ACA: Anterior cerebral artery
CTA: Computed tomography angiography
DW: Diffusion-weighted
ENG: Electronystagmogram
GEM: Gaze-evoked nystagmus
INC: Interstitial nucleus of Cajal
INO: Internuclear ophthalmoplegia
MLF: Medial longitudinal fasciculus
MRI: Magnetic resonance imaging
MS: Multiple sclerosis
NI: Neural integrators
PCA: Posterior cerebral artery
PMT: Paramedian tract
SSN: Seesaw nystagmus
TOAST: Trial of ORG 10172 in Acute Stroke Treatment
